# Behavior of jittering potential before and after impulse blockings: a preliminary study in myasthenia gravis

**DOI:** 10.1007/s13534-024-00401-3

**Published:** 2024-06-14

**Authors:** N. Tuğrul Artuğ

**Affiliations:** Department of Electric, Vocational School of Technical Sciences, Istanbul University-Cerrahpasa, Buyukcekmece, Istanbul, Turkey

**Keywords:** Single-fiber electromyography, Myasthenia gravis, Neuromuscular transmission, Jitter, Impulse blocking, Signal processing

## Abstract

Neuromuscular junction disorders lead to secession of bioelectrical activity transmission between motor nerve endings and muscle fibers. In diseases that are severe enough, impulse blockings are observed. This study aims to reveal the behavior of neuromuscular junction before and after impulse blockings. Fourteen recordings harboring impulse blockings from nine myasthenia gravis (MG) patients were included. Recordings were made from frontalis muscle by using concentric needle electrode during voluntary contraction. One hundred traces were acquired in each session. In addition to well-known jitter parameters, new parameters were calculated such as number of consecutive impulse blocking groups, number of impulse blockings in each group, ratio of maximum number of consecutive impulse blockings to all number of blockings. Graphics were composed to show location change behavior of jittering potential in all traces. For jittering potential, before or after a single impulse blocking the amount of getting further away from trigger peak was greater than getting closer to trigger peak. However, after consecutive impulse blockings the amount of getting closer to trigger peak was greater than the amount of getting further away. The behavior of neuromuscular junction before and after impulse blockings was demonstrated in MG patients. Moreover, new features were extracted for jitter studies. Building models for different diseases according to their impulse blockings may be possible with the developed algorithm.

**Graphical abstract:**

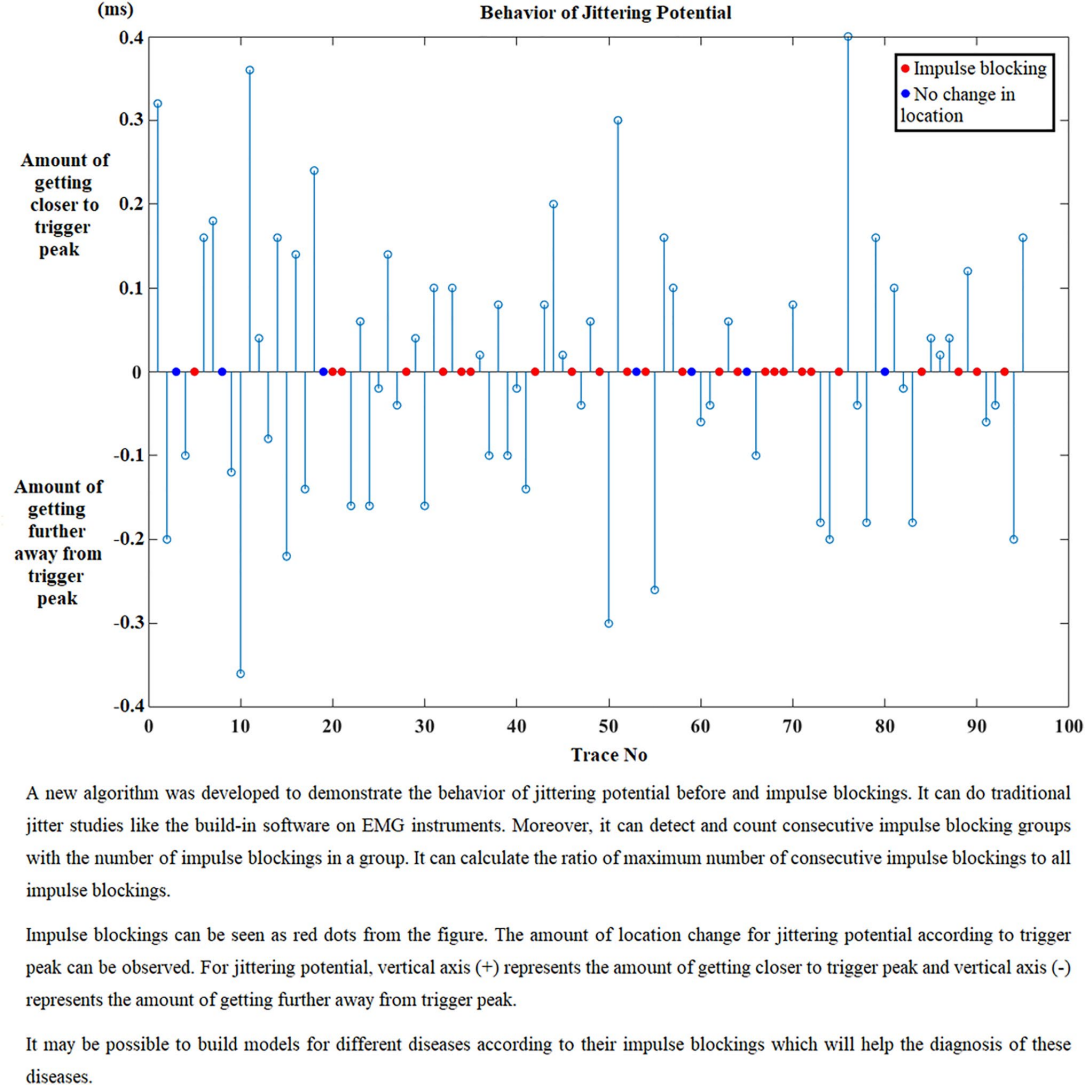

## Introduction

Myasthenia Gravis (MG) is a well-known autoimmune disease that injures post-synaptic side of neuromuscular junction [1, 2]. Repetitive nerve stimulation (RNS) and single fiber EMG (SFEMG) are electrodiagnostic tools for evaluation.

One of the best methods to reveal the neuromuscular junction disorders is SFEMG technique which is developed by Stålberg and Ekstedt [3, 4]. There are several areas for SFEMG to be used, but jitter measurement is the most important one to understand the problems of neuromuscular junction [5]. Jitter can be measured with stimulation method or via voluntary contraction.

In stimulation SFEMG method, jitter represents the variability of measured time between single fiber action potential and axonal stimulation. This calculated time reflects the fluctuation of one end-plate. Under voluntary contraction, jitter shows the fluctuation of two single fiber action potentials. These two action potentials come from two different motor end-plates but from the same motor unit [5, 6].

In order to measure jitter under voluntarily contraction, one action potential is held on the EMG instrument monitor as trigger and the fluctuation of the second action potential which is named as jittering potential is recorded [5]. It is recommended to acquire around 100 consecutive traces for jitter analysis [7]. In every trace, the time difference between the trigger and jittering potential according to their peak value locations is calculated. Then consecutive differences of these time differences are computed. The mean of absolute values of these differences is eventually calculated. This jitter value is named as mean consecutive difference (MCD) [5, 8].

In stimulation method, the measured jitter value is lower because it is produced by only one end-plate. The measured jitter value under voluntary contraction is produced by the contribution of two end-plates, so the jitter value measured with this method is higher when it is compared with the previous one [8, 9].

While recording consecutive traces, sometimes jittering potential is not recorded. Despite a nerve impulse, no single action potential is observed from a motor end-plate. These traces are called as impulse blockings and neuromuscular junction disorders are the reason of this phenomenon [8].

SFEMG technique is the most ideal one for jitter studies certainly, but using a SFEMG electrode is forbidden in some countries because of its drawbacks. It is not a disposable electrode and it must be disinfected to be used again. This process brings fear of infection to mind of some patients. Also, it is expensive and the disinfection process shortens its life [10, 11]. In conclusion, disposable concentric needle electrode (CNE) is the remedy instead of SFEMG electrode [12]. There are successful studies with CNE as well although SFEMG electrode is always the gold standard for the technique [10, 12, 13]. While using CNE for jitter measurement, there are some criteria for recording clean traces [5, 7, 13–15]. These are:

1. Peaks of the traces should have fast rising times.
2. There must be no shoulders or notches on peaks and their shapes must be well-defined.
3. They must keep their shapes same in the next traces.
4. There must be at least 150 µs distance between trigger and jittering potential.
5. A low cut filter with 1 or 2 kHz cut-off frequency should be used to reduce the uptake area of CNE.

Despite its high sensitivity, the SFEMG is less specific comparing to RNS, a test that differentiates post-synaptic involvement from pre-synaptic one relying on the presence of significant decrement or increment, respectively. Moreover, the decrement pattern is also diagnostic for MG if it is “U” or “L” shaped and for Lambert-Eaton myasthenic syndrome (LEMS) by its progressive nature [16–19].

In RNS, the decrement pattern is the reflection of muscle fibers that do not recruit to compound muscle action potential (CMAP) because of neuromuscular blocking. Therefore, RNS tests are diagnostic when enough amounts of neuromuscular impulse blockings are present. This information makes RNS tests more distinctive but less sensitive according to single fiber EMG method. The decrement pattern which is observed through repetitive stimulation is determined by the muscle fibers that are blocked and the muscle fibers which are released from block with the help of recovery mechanisms [16–19].

Significant amplitude changes in a consecutive CMAP raw elicited in response to RNS depends on the percentage of blocking and its pattern is a function of both why the neuromuscular transmission is blocked and how it is overcome [16–18, 20]. However, in SFEMG, although the impulse blockings can also be observed and their percentages are shared along with the jitter value, it is impossible to decide whether it is derived from either pre-synaptic or post-synaptic pathology unless the jitter measurement is performed with axonal stimulation at different stimulation frequencies [21].

One of the most accepted sights of SFEMG performed during voluntary contraction about impulse blockings is that the jitters higher than 100 µs might be accompanied by blocking and in presence of frequent blocking, RNS may be diagnostic for this muscle.

Another important contribution of SFEMG to blocking phenomenon is the term of “axonal–neurogenic blocking” when 2 or more muscle fiber action potentials are blocked together during voluntary contraction as a result of improper conduction of a newly formed axonal sprout that is responsible for reinnervation [22]. Unfortunately, simply noticing the presence of a neuromuscular blocking on jitter measurement is not sufficient and the pathology itself is still obscured by the clouds.

In the previous jitter studies, the change in jitter value before or after blockings on recordings is not a subject which has been discussed much before. Moreover, the fact that the blocking is single or consecutive is a phenomenon that is likely to affect the jitter value. In the conventional EMG instruments, there is no function that demonstrates the behavior of jittering potential directly. Software that will reveal this change may help to reveal how a neuromuscular junction undergoes and quits blocking.

In this preliminary study, the behavior of the jittering potential before and after impulse blockings will be looked closer in a limited number of MG patients. New features for impulse blocking along with the jitter parameter will be presented in graphics by using the developed algorithm. Thus, a new perspective will be formed and it may open a new dimension to understand the behavior of motor end-plates regarding the impulse blockings. Tracking the progression of neuromuscular diseases may also be easier by this method.

The aim of this study is:

- To reveal the behavior of jittering potential before and after impulse blockings in MG patients
- To develop an advanced algorithm that does regular jitter calculations with new features for impulse blockings
- To demonstrate how the impulse blocking becomes prominent with increasing jitter

## Materials and methods

All research was performed in accordance with relevant guidelines and regulations. The study has been approved by the local ethics committee of University of Health Sciences, Bakirkoy Prof. Dr. Mazhar Osman Training and Research Hospital for Psychiatric, Neurologic and Neurosurgical Diseases (2019/348). Written informed consent was obtained from all participants.

### Subjects

Nine generalized MG patients in whom the weakness affects both cranial and extremity muscles (4 female / 5 male) with mean age 51.78 (23–69) accepted to share their recordings to be used for the study. Three patients were seronegative and 6 patients exhibited autoantibodies against the acetylcholine receptor (AchR-ab). Patients who tested positive for the antibody had a mean AchR-ab titer of 6.89 ± 4.57 nmol/L. RNS was performed for confirming electrodiagnostic. All patients revealed jitter values higher than at least 100 µs. There were varying degrees of weakness among all of the MG patients. Three individuals had only ocular symptoms, whereas all of the remaining patients demonstrated generalized weakness on extremities. Nine patients were classified by the criteria of Myasthenia Gravis Foundation of America (MGFA). Two patients were classified as MGFA-3B, 3 were MGFA-2A, 2 were MGFA-1, 1 was MGFA-2B and 1 was MGFA-3A respectively.

### Electrophysiological evaluation

Recordings were made by using a 25 mm, 30 G disposable concentric needle electrode (CNE). Frontalis muscle was examined under voluntarily contraction. Cut off frequency of high pass filter was 1 kHz [5]. Medelec Synergy EMG instrument was used for recording (Natus Medical, USA). The process of acquiring was started when a jittering potential was observed on monitor, time-locked to triggering potential. In each session, 100 traces were recorded. All recordings were made by an expert clinical neurophysiologist. The potential pairs accepted to analysis were recorded during a steady and mild to moderate contraction of the target muscle. Mean consecutive difference (MCD) was calculated as jitter value [8].

Jitter obtained with voluntary contraction represents the fluctuation of neuromuscular transmission at two end-plates. Therefore, the interpotential interval (IPI) change before and after impulse blockings is a common function of these two end-plates [8]. Voluntary contraction SFEMG was preferred in this study rather than the stimulated technique because it was desired to avoid improper stimulation that may lead impulse blocking. Although 20 jitters for each MG patient were calculated, there were some criteria of acceptance for the recordings because of CNE usage. They must be high quality recordings with well-defined peaks both for trigger and jittering potential. All recordings must harbor high number of impulse blockings with high jitter values. As a result of all these constraints, only fourteen jitter recordings were included for analyses to make calculations with the developed algorithm.

### Statistical calculations

SPSS v26 was used to calculate descriptive values for the features. Correlation curves of features were plotted and their approximate equations were given.

### Theory/calculation

Jitter recordings are transferred from EMG instrument to a computer. Raw EMG signal files from the device contain redundant data and they need to be removed by doing pre-processing. The developed algorithm selects the necessary numerical information from raw EMG files, discards letters from recordings and applies noise reduction to the signals in pre-processing stage. If there is motion artifact on any trace among 100 traces, this algorithm lets the user to discard these traces from analysis. The developed algorithm also detects whether the triggering potential switched to another during acquisition and shows these disputable traces in which case the trigger shifting is occurred to examiner on the main window. The decision on these traces about discarding from or keeping in analysis is up to the user.

**Fig. 1:**
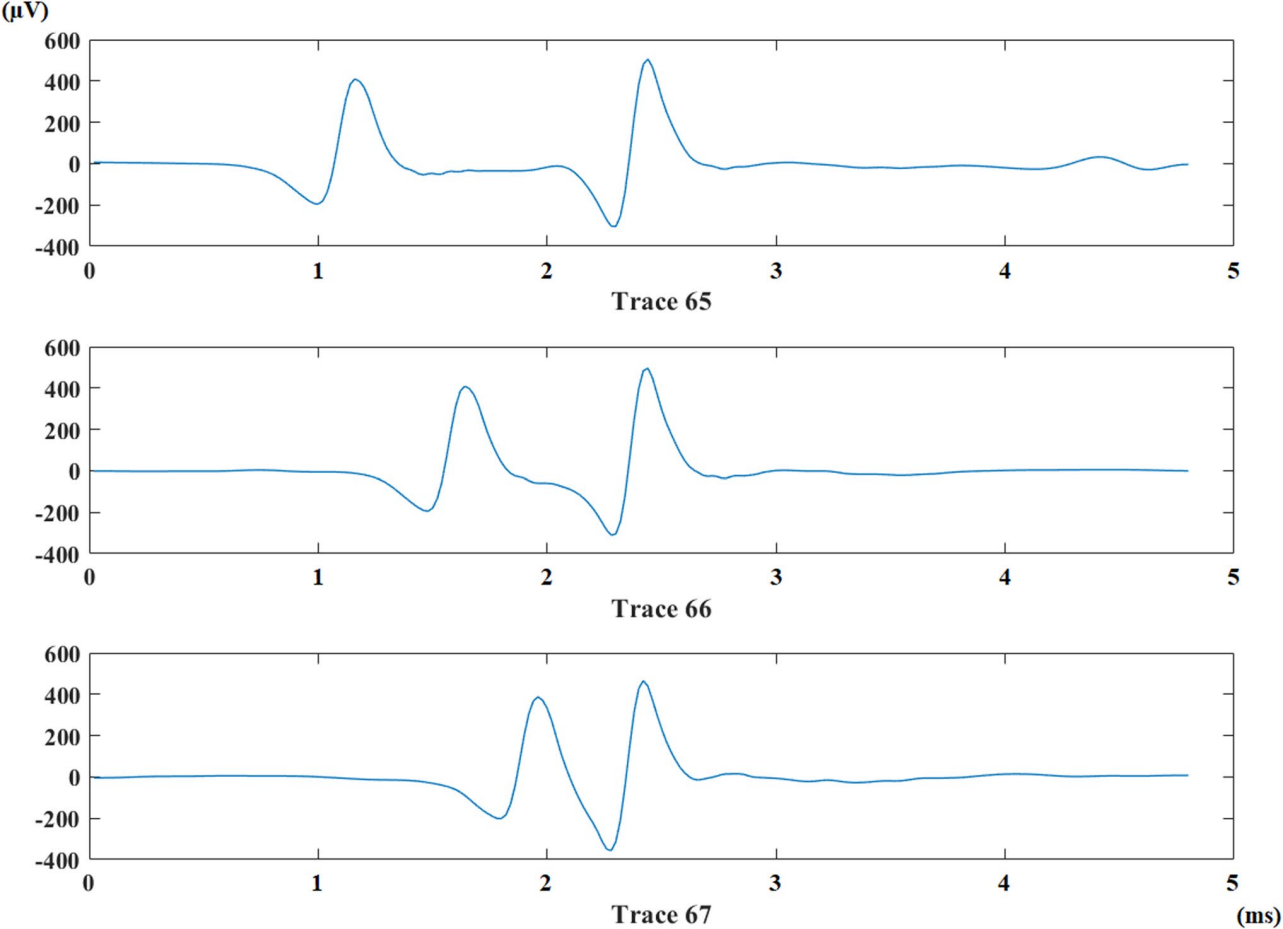
A potential pair from an MG patient. Triggering and jittering potentials are presented in three consecutive traces. Jittering potential is at the left-hand side. (Color figure online)

The trigger peak and jittering potential for jitter analysis are determined from each trace. Their amplitudes and locations are calculated and stored in a matrix for each trace. Jittering potential belonging to an MG patient for three consecutive traces can be seen in Fig. 1.

If there is no jittering potential in a trace, it is marked as an impulse blocking candidate. There must be a ratio lower than 0.2 in terms of amplitude between jittering potential and trigger peak on a trace to be marked as an impulse blocking candidate. This ratio was determined as 0.2 for meeting CNE usage criterion (2) which was about having well-defined peaks. Borderline traces are shown to the user to make a correct impulse blocking marking.

IPI for each trace is calculated. Jitter represents the mean of absolute consecutive differences of IPI values. If there are impulse blockings even though the jitter value is lower than 100 µs, these traces are shown the user to be checked.

**Fig. 2:**
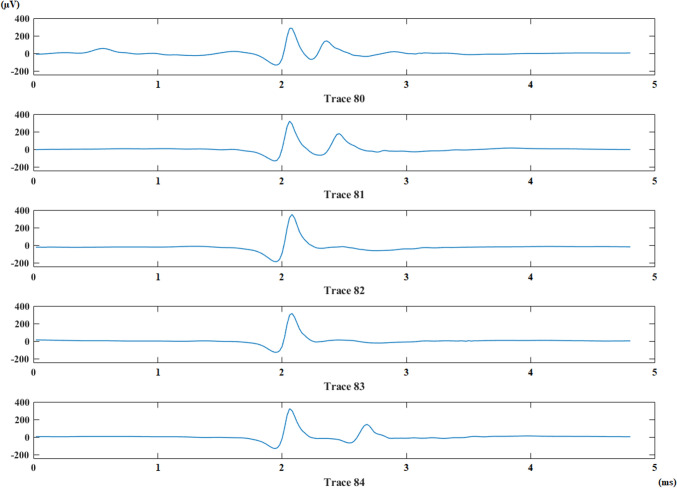
Consecutive impulse blockings in two traces (Traces 82 and 83) for an MG patient. The jittering potential is disappeared in traces 82 and 83. It came back in trace 84 with a longer IPI comparing to that in trace 81. (Color figure online)

The fluctuation of the jittering potential in a recording is determined as the difference between the furthest and the closest location of jittering potential according to trigger peak. This feature is named as interpotential interval range. The number of impulse blockings is calculated along with noticing the presence of consecutive impulse blocking groups. The number of blocks inside of a consecutive block group is also calculated. Maximum number of consecutive blocks and mean value of interpotential interval are calculated as new features for each recording. Consecutive impulse blockings belonging to an MG patient is given in Fig. 2.

**Fig. 3:**
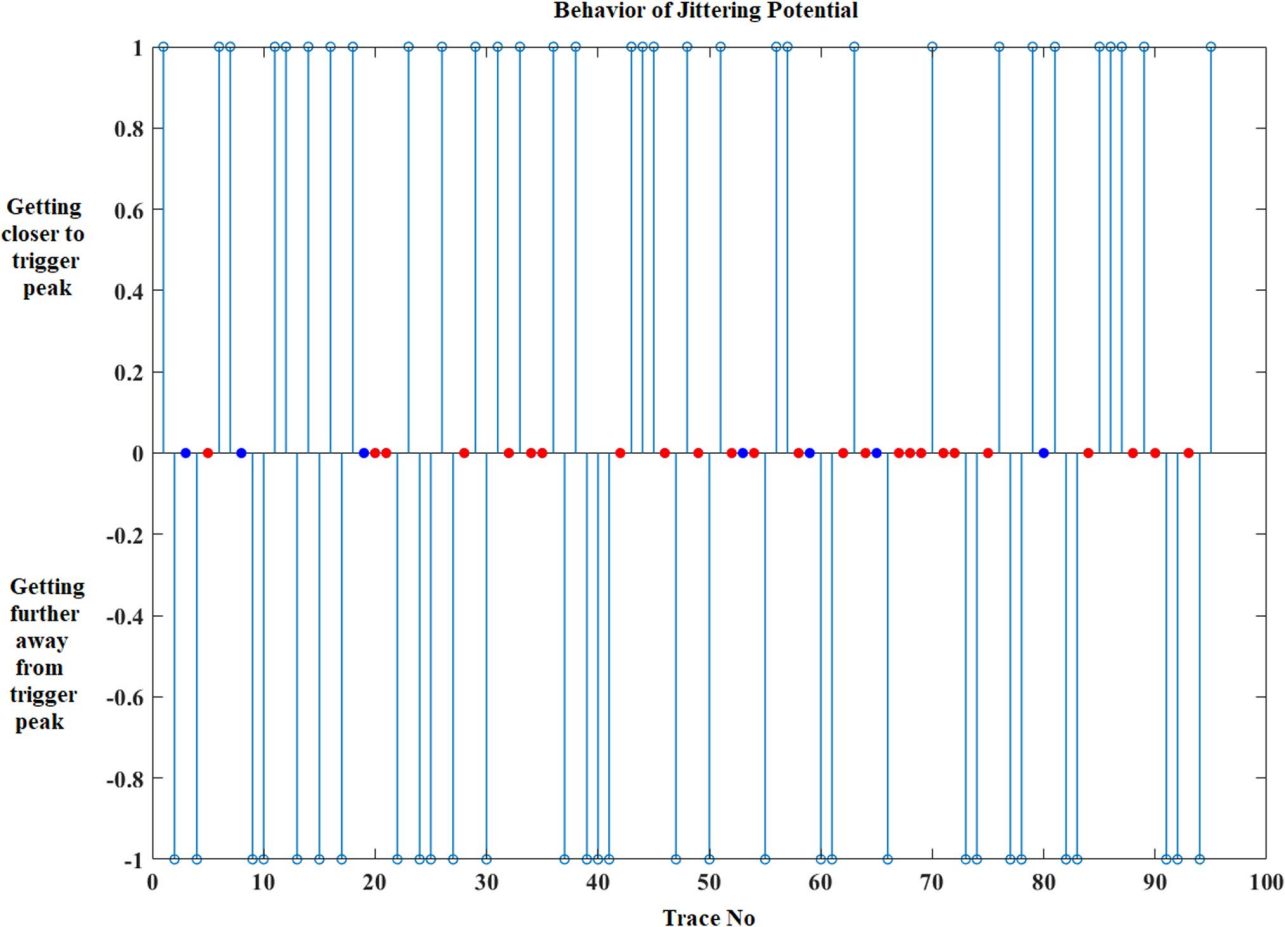
Fluctuation of jittering potential according to the trigger peak for an MG patient (MCD: 122.92 µs). Getting closer to trigger peak + 1, getting further away from trigger peak − 1, impulse blocking is red dot and no change in location is blue dot. (Color figure online)

After the calculation of these parameters, the behavior of jittering potential before and after impulse blockings is shown in graphics. In the first graphic, impulse blockings are marked as zero and changes in the location of jittering potential is marked either “ + 1” or “−1” according to its direction. If the jittering potential’s location does not change at the next trace, then it is coded as zero like impulse blocking and the confusion of displaying both blocking and being steady is solved by decorating them with different colors such as the blue represents “no change in location” and red demonstrates “impulse blocking”. Figure 3 shows the graphic for the fluctuation of jittering potential belonging to an MG patient.

**Fig. 4:**
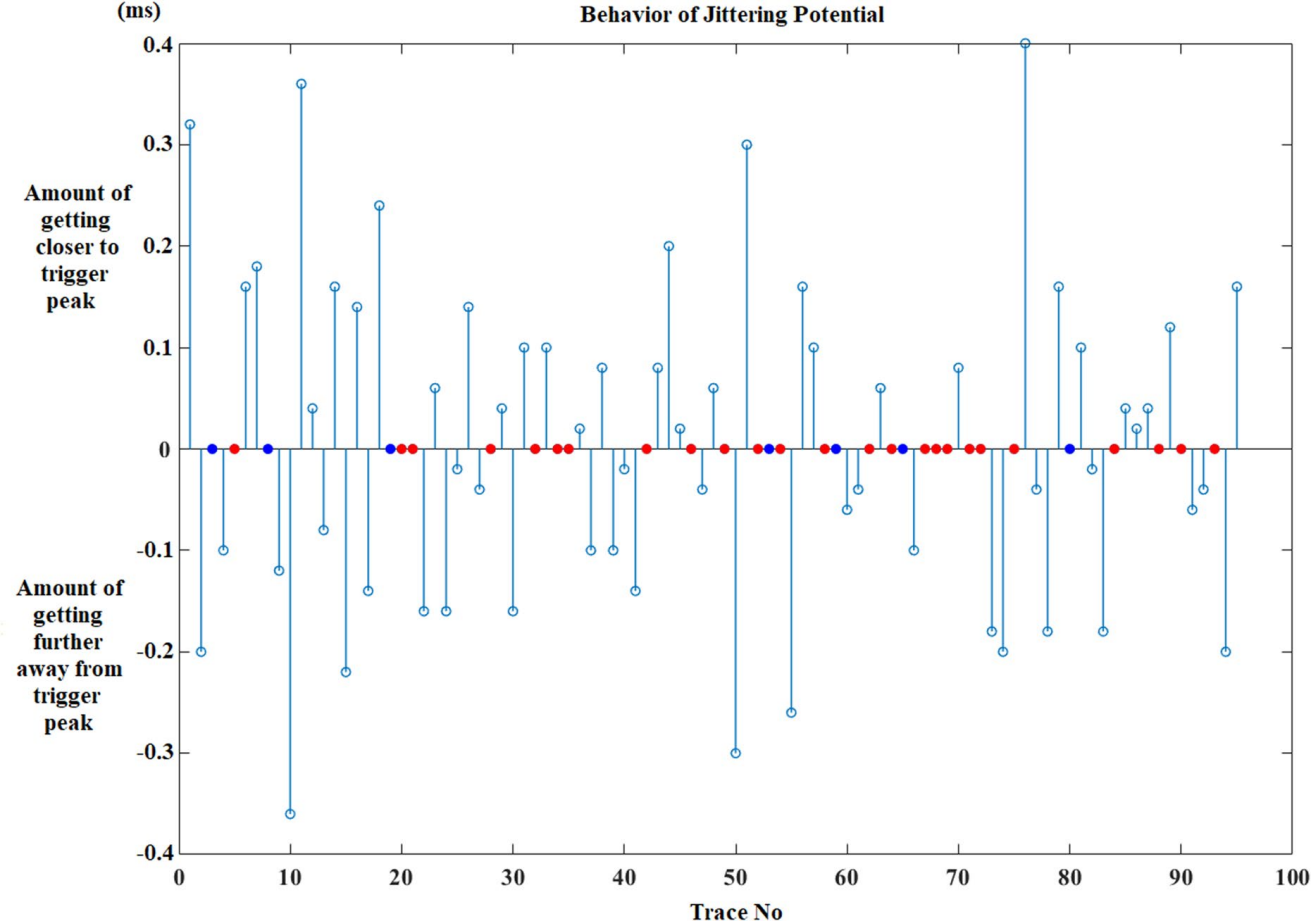
The quantity of direction changes of jittering potential regarding to triggering potential for an MG patient (MCD: 122.92 µs). Getting closer to trigger peak ( +) vertical axis, getting further away from trigger peak (–) vertical axis, impulse blocking is red dot and no change in location of jittering potential is blue dot. (Color figure online)

Second graphic reveals the quantity of direction change that the jittering potential displays (Fig. 4). The information that is harbored in this graphic is more important in demonstrating the behavior of jittering potential before or after impulse blockings in an MG patient.

Finally, the ratio of impulse blockings to all traces and the ratio of maximum number of consecutive impulse blockings to all impulse blockings are calculated and they are displayed to the user. Moreover, the number of block groups is calculated with the number of blocks within block groups and displayed in an array. Last but not least, the number of getting closer to trigger peak and the number of getting further away from trigger peak for jittering potential are demonstrated to the user. One last message appears for the user after analysis for investigating impulse blockings as graphics. If the user desires to check graphics, it obviously prolongs the running time of algorithm.

**Fig. 5:**
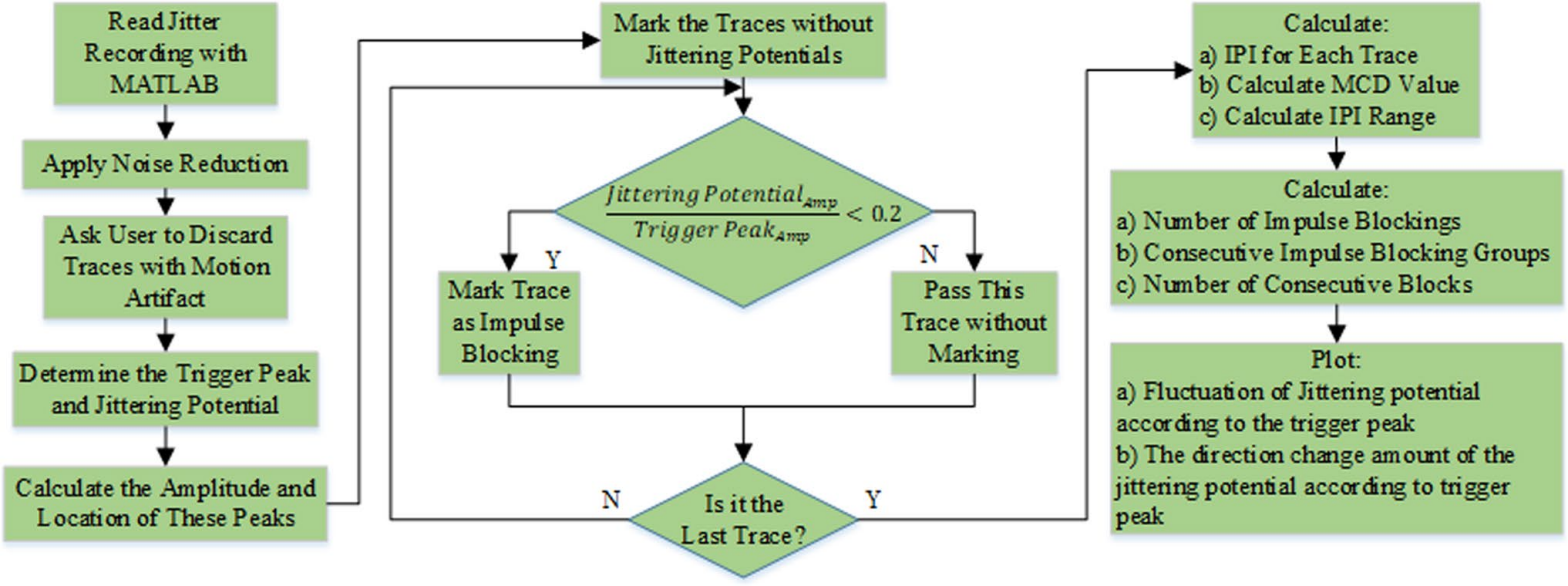
The flowchart for the developed algorithm

The flowchart of the algorithm is shown in Fig. 5.

## Results

Descriptive statistics for the features belonging to MG patients were calculated and they were given in Table 1.

**Table 1:** Calculated values of the features for the jitter recordings

Recording #	Jitter (MCD) (µs)	IPI range (µs)	Mean IPI (µs)	Blocks #	Consecutive block groups #	Max consecutive blocks #	Blocks #/all traces #	Max consecutive blocks #/impulse blockings #
**1**	145.11	1062.5	670.74	11	2	2	0.11	0.18
**2**	123.55	2062.5	1000.29	18	1	2	0.20	0.11
**3**	101.29	458.33	694.3	1	0	0	0.01	0
**4**	144.43	1166.67	891.94	24	5	4	0.24	0.17
**5**	207.11	1291.67	971.92	31	7	4	0.31	0.13
**6**	172.22	833.33	613.78	1	0	0	0.01	0
**7**	173.61	791.67	322.92	40	9	7	0.41	0.18
**8**	122.92	520.83	460.39	25	4	3	0.26	0.12
**9**	107.18	625	354.91	16	1	2	0.16	0.13
**10**	135.76	1875	485.31	29	6	3	0.32	0.10
**11**	250.22	1791.67	743.71	4	0	0	0.04	0
**12**	338.86	1666.67	1005.26	1	0	0	0.01	0
**13**	349.07	1395.83	979.61	4	0	0	0.04	0
**14**	571.43	2333.33	1540.23	69	10	22	0.70	0.32

Mean value of jitter as MCD was found 210.2 ± 130.8 µs for MG patients. Mean value for IPI range was calculated as 1276.79 ± 599.95 µs. Mean IPI value was calculated for MG patients as 766.81 ± 327.09 µs. Number of impulse blockings was 19.57 ± 19.09. Mean value for consecutive impulse blocking groups was calculated as 3.21 ± 3.60. Maximum number of consecutive impulse blockings was calculated 3.5 ± 5.71 as mean value.

**Fig. 6:**
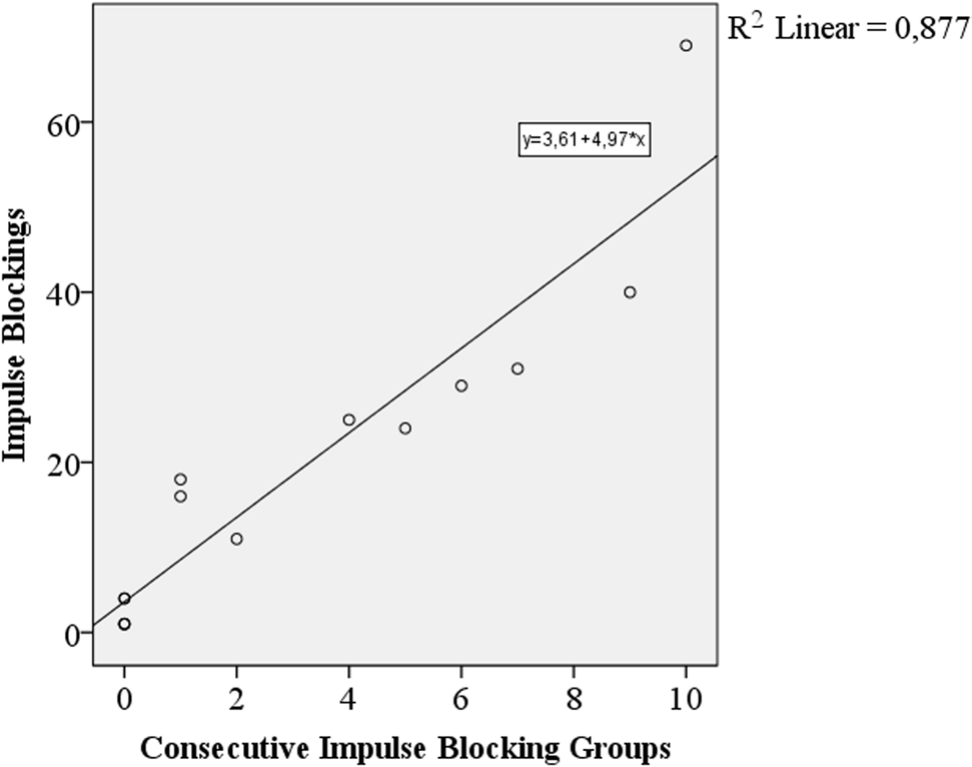
Number of impulse blockings versus number of consecutive impulse blocking groups

When the number of impulse blockings increased, number of consecutive impulse blocking groups was also increased. Figure 6 shows the graphic for number of impulse blockings versus number of consecutive impulse blocking groups.

**Fig. 7:**
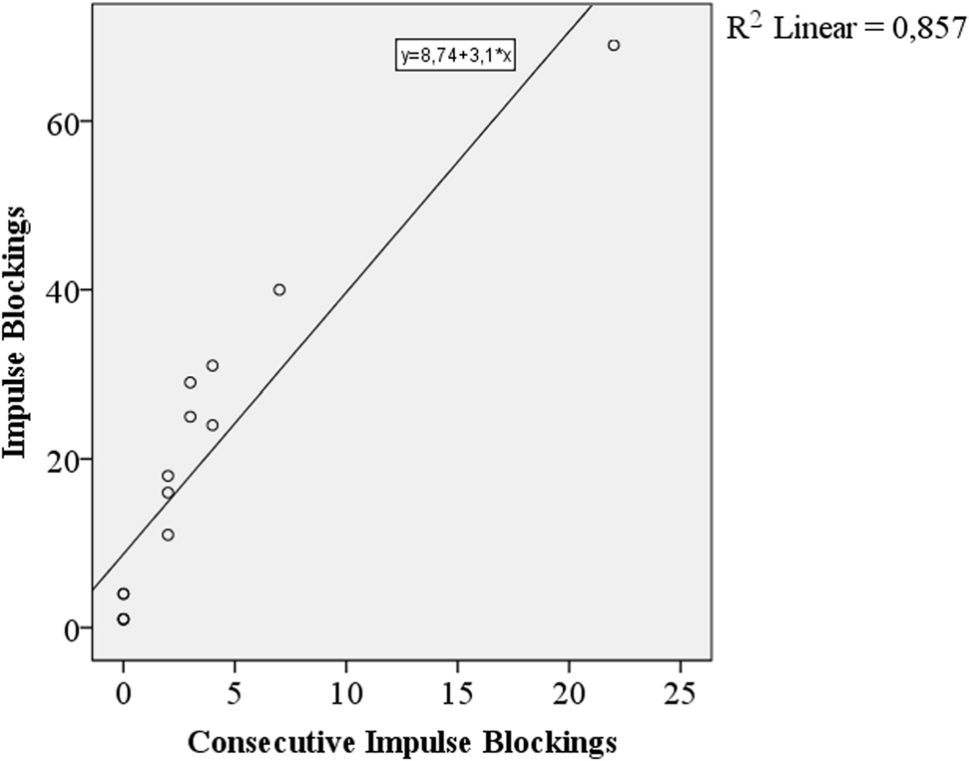
Number of impulse blockings versus maximum number of consecutive impulse blockings

Maximum number of consecutive impulse blockings was also directly proportional to number of impulse blockings. The relation between these two features is given as a graphic in Fig. 7.

**Fig. 8:**
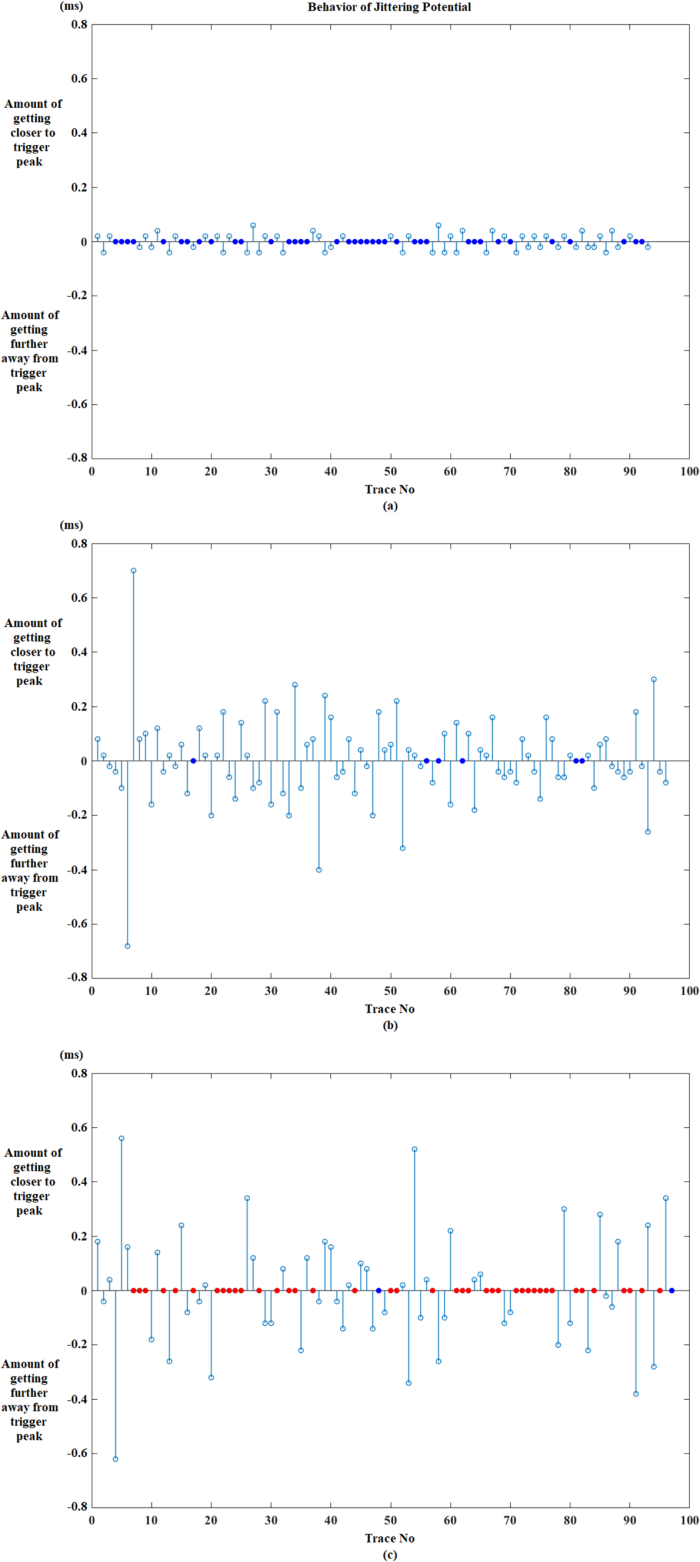
The IPI change according to increasing jitter in a (**a**) healthy individual recording (MCD: 17.7 µs), an (**b**) MG patient recording without impulse blockings (MCD: 111.33 µs) and an (**c**) MG patient recording with impulse blockings (MCD: 173.61 µs). Getting closer to trigger peak ( +) vertical axis, getting further away from trigger peak (–) vertical axis, impulse blocking is red dot and no change in location of jittering potential is blue dot. Also it should be noted that the amount of no change in location of jittering potential is low in the MG patient recording without impulse blockings (**b**) when it is compared with the healthy person recording (**a**). (Color figure online)

The effect of increasing jitter on IPI change was demonstrated in Fig. 8. The first one belongs to a healthy person who has 17.7 µs jitter as MCD. The second one belongs to an MG patient without impulse blockings and the mean jitter value is 111.33 µs for this recording. The last one (MG) has impulse blockings with a 173.61 µs mean jitter value as MCD.

The behavior of jittering potential before and after a single impulse blocking for each recording is given in Table 2. Recordings 3, 6 and 12 (marked with italic values) have only one impulse blocking, so their mean amount of displacement is not added to the calculations of mean value.

**Table 2:** Behavior of jittering potential before and after a single impulse blocking for the jitter recordings

Recording #	The mean amount of displacement before single block (µs)	Behavior before single block	The mean amount of displacement after single block (µs)	Behavior after single block
1	93.75 < < 111.04 > >	Balanced	204.79 > >	Getting further away
2	32.71 < <	Getting closer	55.63 < < 27.71 > >	Balanced
3	83.33 > >	*Getting further away*	41.67 > >	*Getting further away*
4	67.71 < < 187.5 > >	Balanced	263.96 > >	Getting further away
5	267.29 > >	Getting further away	239.58 < < 175 > >	Balanced
6	145.83 < <	*Getting closer*	62.5 < <	*Getting closer*
7	91.67 < < 232.71 > >	Balanced	222.29 < < 150 > >	Balanced
8	104.17 < < 110.21 > >	Balanced	130.21 < <	Getting closer
9	91.15 > >	Getting further away	45 < < 139.79 > >	Balanced
10	260.42 > >	Getting further away	400 < < 166.67 > >	Balanced
11	118.13 < <	Getting closer	177.08 > >	Getting further away
12	62.5 > >	*Getting further away*	–	*No change*
13	583.33 < < 416.67 > >	Balanced	437.5 < < 1000 > >	Balanced
14	333.33 < < 968.75 > >	Balanced	340.21 > >	Getting further away

<  < : Getting closer; >  > : Getting further away

Table 3 shows the behavior of jittering potential before and after a consecutive impulse blocking for each recording. MG recordings 2 and 9 (marked with italic values) have only one consecutive impulse blocking, so their mean amount of displacement is not added to the calculations of mean value. Furthermore, recordings 3, 6, 11, 12 and 13 have not any consecutive impulse blockings.

**Table 3:** Behavior of jittering potential before and after a consecutive impulse blocking for the jitter recordings

Recording #	The mean amount of displacement before consecutive block (µs)	Behavior before consecutive block	The mean amount of displacement after consecutive block (µs)	Behavior after consecutive block
1	187.5 < < 83.33 > >	Balanced	135.42 > >	Getting further away
2	20.83 > >	*Getting further away*	41.47 < <	*Getting closer*
3	–	–	–	–
4	395.83 > >	Getting further away	652.71 < <	Getting c loser
5	348.96 < < 527.71 > >	Balanced	487.5 < <	Getting closer
6	–	–	–	–
7	145.83 < < 156.25 > >	Balanced	229.17 > >	Getting further away
8	93.75 < < 104.17 > >	Balanced	52.08 < < 177.08 > >	Balanced
9	104.17 < <	*Getting closer*	–	*No Change*
10	385.42 < < 72.92 > >	Balanced	598.96 > >	Getting further away
11	–	–	–	–
12	–	–	–	–
13	–	–	–	–
14	305.63 > >	Getting further away	718.75 < <	Getting closer

<  < : Getting closer; >  > : Getting further away

The mean amount of getting closer to trigger peak and getting further away from trigger peak for jittering potential was calculated. If there is only one single impulse blocking or consecutive impulse blocking, it is not included in the calculation of mean value.

The mean value for getting closer of jittering potential to trigger peak before a single impulse blocking was 178.10 µs for MG patients. Same value for getting further away was 293.97 µs.

After a single impulse blocking, the mean value for getting closer of jittering potential to trigger peak was 218.60 µs for MG patients. The mean getting further away amount was calculated as 264.52 µs.

The mean value for getting closer of jittering potential to trigger peak before a consecutive impulse blocking was 232.29 µs for MG patients. Same value for getting further away was 235.12 µs.

After a consecutive impulse blocking, the mean value for getting closer of jittering potential to trigger peak was 477.76 µs for MG patients. The mean getting further away amount was calculated as 285.16 µs.

Lastly, a comparison in tabular form for traditional jitter studies in chronological order such as measuring jitter value in MCD and/or calculating percentage of impulse blockings is given in Table 4. Obviously, there are lots of jitter studies in the literature, so the studies in Table 4 were selected according to the following criteria. All studies must have MG patients in their dataset and jitter value must be given as MCD. Investigated muscle, activation technique or needle should be same with one in the current study. After all, 12 studies were included in the table. Please note that for some studies, standard deviation value for jitter was not shared and impulse blocking was not studied or present.

**Table 4:** The summary of calculated parameters in jitter studies from different references

Study and year	Investigated muscle	Activation technique	Jitter value (μs) [Mean ± SD]	Block (%)	Needle
Sanders & Howard, 1986 [23]	FR	Voluntarily	76	20	SFEMG
Sanders & Stalberg, 1996 [8]	EDC	Voluntarily	88	28	SFEMG
Ertaş et al. 2000 [12]	EDC	Voluntarily	65.3 ± 51	–	CN
Valls-Canals et al. 2003 [24]	FR	Stimulated	43.85 ± 25.18	–	SFEMG
Farugia et al., 2009 [15]	OO	Voluntarily	55.1 ± 36.6	20	CN
Farugia et al. 2009 [15]	EDC	Voluntarily	58.8 ± 42.5	21	CN
Kouyoumdjian et al. 2011 [7]	FR	Stimulated	63.3	38.3	CN
Machado et al. 2017 [25]	OO	Voluntarily	60.3 ± 49.3	13	CN
Abraham et al. 2017 [26]	FR	Voluntarily	93	–	SFEMG
Sciacca et al. 2018 [27]	EDC	Voluntarily	58.9 ± 18.8	6.2	CN
Sirin et al. 2018 [28]	FR	Voluntarily	89.4 ± 46.1	–	CN
Kouyoumdjian et al. 2020 [29]	FR	Stimulated	66.3	20.9	CN
Artug, 2024	FR	Voluntarily	210.2 ± 130.8	20.19	CN

*FR*, frontalis; *EDC*, extensor digitorum communis; *OO*, orbicularis oculi; *SFEMG*, single fiber electromyography; *CN*, concentric needle

## Discussion

The developed software can do traditional jitter studies like the build-in software on EMG instruments. Besides, it has some other advantages such as detecting impulse blockings and consecutive impulse blocking groups, counting the number of impulse blockings, calculating the ratio of impulse blockings to all traces, calculating the ratio of consecutive impulse blockings to all impulse blockings, plotting graphics to show the behavior of jittering potential before and after impulse blockings. These graphics reveal the behavior trend of jittering potential. It is possible to observe the direction change of jittering potential and the amount of the direction change of it according to trigger peak. If there is a borderline trace which has values near blocking threshold value in a recording, this trace is shown to user for deciding on marking it as an impulse blocking or not. The related trace in that recording is displayed with previous and next traces to make an easier decision for the user. It is also possible to use the algorithm fully automatic with a significant evaluation performance. The algorithm completes the analyses approximately in less than 1 min with 3.40 GHz CPU, 8 GB DDR3 RAM configured PC.

When the results are examined, before or after a single impulse blocking, getting further away of jittering potential from trigger peak is greater than getting closer in terms of amount in MG patients.

For jittering potential, the amount of getting closer to trigger peak is greater than the amount of getting further away from trigger peak after consecutive impulse blockings. Before consecutive impulse blockings, it is not possible to mention about the behavior for MG patients. The values about getting closer or getting further away for jittering potential to trigger peak are similar.

The reason behind the unpredictable behavior before consecutive impulse blockings can be the nature of jitter itself which is recorded under voluntarily contraction because this jitter represents the fluctuation of two end-plates [8]. If both of the neuromuscular junctions are not blocked, these two potentials can move randomly.

On the other hand, after consecutive impulse blockings, the neuromuscular junction that produces jittering potential takes an opportunity to have some rest. Thus, this opportunity may increase the acetylcholine amount which is released from pre-synaptic nerve terminal and end-plate potential can pass over the threshold to trigger the action potential again as a result of restored safety factor which is a function of both acetylcholine amount and available acetylcholine receptors. Consequently, the reason of getting closer to trigger peak after consecutive impulse blockings is mostly because of the high safety factor of the neuromuscular junction that triggers jittering potential. If jittering potential is getting further away from trigger peak after impulse blocking, previous jittering potential may be below the safety factor, but then it may reaches the critical level barely. Thus, jittering potential appears eventually even if it is late.

It is obvious that this study is done by using voluntarily contraction, so the variability of IPI is determined by the fluctuation of the two end-plate potentials. If the neuromuscular junction that produces the trigger potential is blocked, the monitor of the EMG instrument is not triggered and the impulse blocking cannot be noticed by the examiner. This variable which cannot be measured may contribute to the unpredictable behavior of IPI value between potential pairs before or after impulse blockings. Moreover, if two end-plates are blocked simultaneously, it cannot be noticed by the examiner too.

Stimulated SFEMG reflects the IPI change of an individual end-plate better. However, if an impulse blocking occurs with improper stimulation, it can be hard to distinguish this from a real neuromuscular junction failure. If the single fiber potential does not appear on monitor in some traces while the end-plate under investigation shows normal jitter at previous recordings, it can easily be interpreted as a situation related with stimulation technique. Nonetheless, during recording of a neuromuscular junction with defected transmission which has high jitter and variable IPI value, absence of jittering potential in some traces may either be caused by neuromuscular junction blocking or improper stimulation. In such a case, the detection of IPI change before and after blocking cannot be considered independent of technical difficulties.

Theoretically, it is impossible to ascribe a given IPI to one of the two involved end-plates in voluntary SFEMG. Hence, the potential pairs are recorded from the same motor unit of the same muscle. It can be assumed that these two neuromuscular junctions both suffer from the same pathophysiology, but the severity of the pathophysiology can be different. Therefore, it seems plausible to use voluntary SFEMG for studying blockings while keeping the drawbacks in mind.

It is possible to make transition between the stimulation technique jitter value and the voluntarily contraction jitter value. The jitter of two end-plates can be formulated. First, the jitter of each one with the stimulation technique is measured. Then the following equation is used to calculate combined jitter of two end-plates [30]

1 $$ Combined\,\, Jitter = \sqrt {Jitter_{A}^{2} + Jitter_{B}^{2} } $$

For a single neuromuscular junction, minimum jitter value can be 5 µs via axonal stimulation [5].

It is expected that if the number of impulse blockings increases, the probability of observing consecutive impulse blockings also increases. The results validate this suggestion. Number of impulse blockings is directly proportional with number of consecutive impulse blocking groups and maximum number of consecutive impulse blockings.

If the jitter value is greater than 100 µs, the probability of impulse blocking increases. In MG which is a post-synaptic disease, if low rate RNS test is done after observing high number of blockings, primary quanta storage depletes and a decrement pattern is observed. Then, secondary quanta storage replenishes the primary one and end-plate potential demonstrates an increment pattern again [31]. For this reason, after observing high jitter and consecutive impulse blockings, low rate RNS test can help to diagnose the disease with its “U” or “L” shaped graphic pattern [17].

The amplitude decrement of CMAP in RNS for MG patients represents the increment of number of muscle fibers that are not contributing to the response with consecutive stimuli. The increment of this number is probably explained as that the new neuromuscular junctions which are blocked are joined to the other neuromuscular junctions that are demonstrating consecutive impulse blockings. The slight amplitude increment of CMAP after stimuli (“U” shaped decrement) is depended on recover from the impulse blocking of some neuromuscular junctions. If the CMAP amplitude remains same at the decreased level after repetitive stimuli (“L” shaped decrement), it indicates that the number of blocked neuromuscular junctions remains same. On this basis, the sustainable presence of impulse blockings on consecutive traces that are recorded under voluntarily contraction with SFEMG method is a better inspection that predicts the decrement response of RNS test.

In the study of Valls-Canals and his colleagues [24], the percentage of impulse blockings was presented and they made their recordings from MG patients with stimulation technique while ours was voluntarily contraction. In stimulation technique, jitter value represents the transmission variability of one motor end-plate. That’s why the jitter value is lower [8, 32]. It gives an advantage to examiner to decide which stimulation frequency will be chosen like the RNS test. In the recordings under voluntarily contraction which harbor a high number of consecutive impulse blockings, the neuromuscular junction that produces the trigger potential can be blocked frequently. For this reason, doing recordings with stimulated technique may be helpful. However, improper stimulation may affect jitter value and also this can reflect as impulse blocking to the recordings because of the muscle fiber is not stimulated.

In their study [24], they found the mean jitter value for frontalis muscle as 43.85 ± 25.18 µs. The same value was calculated as 210.2 ± 130.8 µs with the developed algorithm of current study. They also reported the mean value for number of impulse blockings as 2.18 ± 3.02 for the same muscle. In the current study, the mean value for impulse blockings was calculated as 19.57 ± 19.09. The difference for this value between these two studies might be because of the recordings in the dataset of the current study. These recordings were revealed high jitter values and they were preferred among the recordings that contained impulse blockings.

In the year of 2017, Machado and the others [25] made their recordings from orbicularis oculi muscle of MG patients and normal individuals. They used CNE and preferred voluntarily contraction too as in the current study. The calculated jitter value was 60.3 ± 49.3 µs. The mean value for IPI range was 1000 ± 200 µs, while it was 1276.79 ± 599.95 µs for the same feature in the current study. They shared the ratio of impulse blockings as 13% for MG patients. In the current study, the ratio of impulse blockings to all traces for MG patients was 20.19%. This value was higher in the current study because the mean jitter value as MCD was also higher than their study. The increment in jitter value reflects to the probability of occurrence for impulse blockings. It should be noted that the recording muscle was also different than the preferred muscle in current study.

Sirin et al. made a study on MG patients in 2018 [28]. Thirty MG patients enrolled to the study. Twenty two of them were classified as MGFA I–II while the other eight were classified as MGFA III–IV. Investigated muscle was frontalis muscle like in the current study. They recorded at least 60 traces by using CNE under voluntary contraction. Jitter value was shared with mean value 89.4 ± 46.1 µs for MGFA III–IV classified patients. On the other hand, same value was 210.2 ± 130.8 µs in the current study. Any information on impulse blockings was not shared, so a direct comparison was not possible for this feature. Patient cohort was the reason behind this high jitter value in the current study. The recordings were chosen intentionally to have high jitter values at least 100 µs. Thus, the recordings harbored impulse blockings.

The variation in IPI was taken into account as the reflection of neuromuscular junction transmission. The recordings were made during steady contraction of the muscle under investigation. For this reason, MCD was calculated. If the contraction level had been variable, MSD would have been calculated [9]. However, sorting the traces according to firing frequencies of motor units changes their order, so demonstrating IPI behavior could not be possible by this way.

The behavior of jittering potential before and after impulse blockings in myasthenia patients has not been studied thoroughly. The developed algorithm is promising to demonstrate the behavior of jittering potential in recordings according to impulse blockings. The build-in software on EMG instruments does not have flexibility while the developed algorithm in the current study has. It has extra talents and lets the user to have control over some decisions on recordings. The developed algorithm can do all jitter analysis under 2 (usually < 1) minutes including manual adjustments.

An option was also added to show the impulse blockings to the user after completion of analysis. Thus, mislabeling and misrecognition of trigger potential and jittering potential can be examined and confirmed visually by the examiner. It should not be forgotten that if this option is selected, the execution time of the algorithm prolongs.

The proposed algorithm may not help diagnosis of MG disease. Neuromuscular junction which has pre-synaptic and post-synaptic sides is a specialized structure that conducts information according to graded response philosophy. In this respect, it may open a door to quantitative evaluation of all kinds of components which can contribute to the physiological transmission of chemical synapses.

On the other hand, the number of recordings included in this study that has high jitter with impulse blockings is low. In addition, there are not any recordings which belong to a LEMS patient because it is very rare to encounter a patient with this disease. A study demonstrated that the prevalence of this disease as 2.3 per million [33]. Lastly, the voluntarily contraction technique does not reveal trigger potential block or the block of both end-plates.

For the future studies, dataset will expand with new recordings and new patient groups will be added. It is planned to have recordings which belong to pre-synaptic diseases too. They all will be compared with the others by using the developed algorithm. It may be possible to determine a model for impulse blockings in different diseases that affecting the neuromuscular junction in future studies. A graphic unit interface (GUI) will be developed to simplify the usage of this software for neurophysiologists.

## Conclusion

A new algorithm was developed to demonstrate the behavior of jittering potential before and after impulse blockings in jitter studies. Some new features intended for impulse blockings were extracted. Consecutive impulse blockings were also considered. The constituted graphics were helpful to differentiate the behavior pattern before and after impulse blockings. Besides, the algorithm let the user to decide on critical traces by showing previous and next traces from that recording while marking that trace as an impulse blocking or not.

A remarkable finding was observed after consecutive impulse blockings occurred in MG patients. The amount of getting closer for jittering potential to trigger peak was greater than the amount of getting further away.

The results revealed direct proportion between the number of impulse blockings and maximum number of consecutive impulse blockings. The same relation was also present between the number of impulse blockings and the number of consecutive impulse blocking groups too.

## References

[1] Brown RH, Cannon SC, Rowland LP. Diseases of the nerve and motor unit. In: Kandel ER, Schwatz JH, Jessell TM, Siegelbaum SA, Hudspeth AJ, editors. Principles of neural science. 5th ed. New York: McGraw-Hill; 2012. p. 314–8.

[2] Drachman DB. Myasthenia gravis. N Engl J Med. 1994;330(25):1797–810. 10.1056/NEJM199406233302507. 8190158 10.1056/NEJM199406233302507

[3] Ekstedt J. Human single muscle fiber action potentials. Extracellular recording during voluntary and chemical activation. with some comments on end-plate physiology and on the fiber arrangement of the motor unit. Acta Physiol Scand Suppl. 1964;226:1–96. 14150641

[4] Stålberg EV, Trontelj JV. Single fiber electromyography: Studies in healthy and diseased muscle. 2nd ed. New York: Raven Press; 1994.

[5] Sanders DB, Arimura K, Cui L, Ertaş M, Farrugia ME, Gilchrist J, Kouyoumdjian JA, Padua L, Pitt M, Stålberg E. Guidelines for single fiber EMG. Clin Neurophysiol. 2019;130(8):1417–39. 10.1016/j.clinph.2019.04.005. 31080019 10.1016/j.clinph.2019.04.005

[6] Kouyoumdjian JA, Stålberg EV. Concentric needle single fiber electromyography: comparative jitter on voluntary-activated and stimulated extensor digitorum communis. Clin Neurophysiol. 2008;119(7):1614–8. 10.1016/j.clinph.2008.03.008. 18455474 10.1016/j.clinph.2008.03.008

[7] Kouyoumdjian JA, Fanani AC, Stålberg EV. Concentric needle jitter on stimulated frontalis and extensor digitorum in 20 myasthenia gravis patients. Muscle Nerve. 2011;44(6):912–8. 10.1002/mus.22203. 22102462 10.1002/mus.22203

[8] Sanders DB, Stålberg EV. AAEM minimonograph #25: single-fiber electromyography. Muscle Nerve. 1996;19(9):1069–83. 10.1002/(SICI)1097-4598(199609)19:9%3c1069::AID-MUS1%3e3.0.CO;2-Y. 8761262 10.1002/(SICI)1097-4598(199609)19:9<1069::AID-MUS1>3.0.CO;2-Y

[9] Stålberg E, Sanders DB, Kouyoumdjian JA. Pitfalls and errors in measuring jitter. Clin Neurophysiol. 2017;128(11):2233–41. 10.1016/j.clinph.2017.09.001. 29017138 10.1016/j.clinph.2017.09.001

[10] Sarrigiannis PG, Kennett RP, Read S, Farrugia ME. Single-fiber EMG with a concentric needle electrode: validation in myasthenia gravis. Muscle Nerve. 2006;33(1):61–5. 10.1002/mus.20435. 16175626 10.1002/mus.20435

[11] Benatar M, Hammad M, Doss-Riney H. Concentric-needle single-fiber electromyography for the diagnosis of myasthenia gravis. Muscle Nerve. 2006;34(2):163–8. 10.1002/mus.20568. 16642500 10.1002/mus.20568

[12] Ertaş M, Baslo MB, Yildiz N, Yazici J, Oge AE. Concentric needle electrode for neuromuscular jitter analysis. Muscle Nerve. 2000;23(5):715–9. 10.1002/(sici)1097-4598(200005)23:5%3c715::aid-mus8%3e3.0.co;2-v. 10797394 10.1002/(sici)1097-4598(200005)23:5<715::aid-mus8>3.0.co;2-v

[13] Kouyoumdjian JA, Stålberg EV. Reference jitter values for concentric needle electrodes in voluntarily activated extensor digitorum communis and orbicularis oculi muscles. Muscle Nerve. 2008;37(6):694–9. 10.1002/mus.21043. 18506720 10.1002/mus.21043

[14] Stålberg EV, Sanders DB. Jitter recordings with concentric needle electrodes. Muscle Nerve. 2009;40(3):331–9. 10.1002/mus.21424. 19705424 10.1002/mus.21424

[15] Farrugia ME, Weir AI, Cleary M, Cooper S, Metcalfe R, Mallik A. Concentric and single fiber needle electrodes yield comparable jitter results in myasthenia gravis. Muscle Nerve. 2009;39(5):579–85. 10.1002/mus.21151. 19260051 10.1002/mus.21151

[16] Tim RW, Sanders DB. Repetitive nerve stimulation studies in the Lambert-Eaton myasthenic syndrome. Muscle Nerve. 1994;17(9):995–1001. 10.1002/mus.880170906. 8065402 10.1002/mus.880170906

[17] Baslo MB, Deymeer F, Serdaroglu P, Parman Y, Ozdemir C, Cuttini M. Decrement pattern in Lambert-Eaton myasthenic syndrome is different from myasthenia gravis. Neuromuscul Disord. 2006;16(7):454–8. 10.1016/j.nmd.2006.05.009. 16806929 10.1016/j.nmd.2006.05.009

[18] Ozdemir C, Young RR. Electrical testing in myasthenia gravis. Ann N Y Acad Sci. 1971;183:287–302. 10.1111/j.1749-6632.1971.tb30759.x. 5287827 10.1111/j.1749-6632.1971.tb30759.x

[19] AAEM quality assurance committee. American association of electrodiagnostic medicine. Literature review of the usefulness of repetitive nerve stimulation and single fiber EMG in the electrodiagnostic evaluation of patients with suspected myasthenia gravis or Lambert-Eaton myasthenic syndrome. Muscle Nerve. 2001;24(9):1239–47. 10.1002/mus.1140. 11494281 10.1002/mus.1140

[20] Chiou-Tan FY, Gilchrist JM. Repetitive nerve stimulation and single-fiber electromyography in the evaluation of patients with suspected myasthenia gravis or Lambert-Eaton myasthenic syndrome: Review of recent literature. Muscle Nerve. 2015;52(3):455–62. 10.1002/mus.24745. 26109387 10.1002/mus.24745

[21] Stålberg E, Trontelj JV, Schwartz MS. Single-muscle-fiber recording of the jitter phenomenon in patients with myasthenia gravis and in members of their families. Ann N Y Acad Sci. 1976;274:189–202. 10.1111/j.1749-6632.1976.tb47685.x. 1066986 10.1111/j.1749-6632.1976.tb47685.x

[22] Stålberg E. Clinical electrophysiology in myasthenia gravis. J Neurol Neurosurg Psychiatry. 1980;43(7):622–33. 10.1136/jnnp.43.7.622. 6249895 10.1136/jnnp.43.7.622 PMC490629

[23] Sanders DB, Howard JF Jr. AAEE minimonograph #25: Single-fiber electromyography in myasthenia gravis. Muscle Nerve. 1986;9(9):809–19. 10.1002/mus.880090904. 3785290 10.1002/mus.880090904

[24] Valls-Canals J, Povedano M, Montero J, Pradas J. Stimulated single-fiber EMG of the frontalis and orbicularis oculi muscles in ocular myasthenia gravis. Muscle Nerve. 2003;28(4):501–3. 10.1002/mus.10426. 14506723 10.1002/mus.10426

[25] Machado FCN, Kouyoumdjian JA, Marchiori PE. Diagnostic accuracy of concentric needle jitter in myasthenia: prospective study. Muscle Nerve. 2017;55(2):190–4. 10.1002/mus.25229. 27348087 10.1002/mus.25229

[26] Abraham A, Breiner A, Barnett C, Katzberg HD, Bril V. Recording fewer than 20 potential pairs with SFEMG may suffice for the diagnosis of myasthenia gravis. J Clin Neurophysiol. 2017;34(5):408–12. 10.1097/WNP.0000000000000402. 28650411 10.1097/WNP.0000000000000402

[27] Sciacca G, Reggio E, Mostile G, Nicoletti A, Drago F, Salomone S, Zappia M. Clinical and CN-SFEMG evaluation of neostigmine test in myasthenia gravis. Neurol Sci. 2018;39(2):341–5. 10.1007/s10072-017-3194-0. 29330628 10.1007/s10072-017-3194-0

[28] Sirin NG, Kocasoy Orhan E, Durmus H, Deymeer F, Baslo MB. Repetitive nerve stimulation and jitter measurement with disposable concentric needle electrode in newly diagnosed myasthenia gravis patients. Neurophysiol Clin. 2018;48(5):261–7. 10.1016/j.neucli.2018.01.003. 29490884 10.1016/j.neucli.2018.01.003

[29] Kouyoumdjian JA, Paiva GP, Stålberg E. Concentric needle jitter in 97 myasthenia gravis patients. Front Neurol. 2020;11:600680. 10.3389/fneur.2020.600680. 33281737 10.3389/fneur.2020.600680 PMC7691317

[30] Stålberg EV, Trontelj JV, Sanders DB. Jitter and impulse blocking. In: Stålberg EV, Trontelj JV, Sanders DB, editors. Single fiber EMG. 3rd ed. Sweden: Edshagen Publishing; 2010. p. 46–84.

[31] Shapiro BE, Preston DC. Repetitive nerve stimulation and exercise testing. Phys Med Rehabil Clin N Am. 2003;14(2):185–206. 10.1016/s1047-9651(02)00129-8. 12795512 10.1016/s1047-9651(02)00129-8

[32] Jabre JF, Chirico-Post J, Weiner M. Stimulation SFEMG in myasthenia gravis. Muscle Nerve. 1989;12(1):38–42. 10.1002/mus.880120108. 2747735 10.1002/mus.880120108

[33] Wirtz PW, Nijnuis MG, Sotodeh M, Willems LN, Brahim JJ, Putter H, Wintzen AR, Verschuuren JJ, Dutch Myasthenia Study Group. The epidemiology of myasthenia gravis, Lambert-Eaton myasthenic syndrome and their associated tumours in the northern part of the province of South Holland. J neurol. 2003;250(6):698–701. 10.1007/s00415-003-1063-7. 12796832 10.1007/s00415-003-1063-7

